# Supplementary material for: A zinc oxide resonant nano-accelerometer with ultra-high sensitivity
Source: Nat Commun. 2024 May 31;15:4651. doi: 10.1038/s41467-024-49145-9 (PMC11143280; doi:10.1038/s41467-024-49145-9)
Supplement: Supplementary file 1 — Supplementary Information [file 41467_2024_49145_MOESM1_ESM.pdf]

Supplementary Materials

A zinc oxide resonant nano-accelerometer with ultra-high sensitivity

Pengfei Xu, Dazhi Wang, Jianqiao He, Yichang Cui, Liangkun Lu, Yikang Li, Xiangji Chen, Chang Liu, Liujia Suo, Tongqun Ren, Tiesheng Wang and Yan Cui*

Note S1. Principle of the resonant accelerometer

Resonant accelerometers use the characteristic of frequency drift of sensing elements under axial force to detect acceleration. The commonly used theoretical research methods for sensitive beams of resonant accelerometers are the Euler-Bernoulli beam theory and the Timoshenko beam theory. The Timoshenko beam theory considers the influence of beam cross-sectional rotation and shear deformation on the basis of the Euler-Bernoulli theory, and has significant improvement in the analysis results for non-slim beams and high-order modes. For slim beams with length-to-diameter ratio greater than 100 and ignoring the effects of shear and rotation, the Euler-Bernoulli beam theory is more convenient in solving. Based on the vibration characteristics of Euler-Bernoulli beams, this section solves the resonant frequency of ZnO nanobeams and obtains the force sensitivity expression of the resonant accelerometer, and analyzes the nonlinear factors affecting the sensitivity of the resonant beam.

Resonant frequency of nanobeam

The ZnO nanobeam in the accelerometer with a large aspect ratio conforms to the typical Euler-Bernoulli beam. According to the classical bending vibration theory of Euler-Bernoulli beam, the free vibration equation of ZnO nanobeam shown in Figure S1 is:

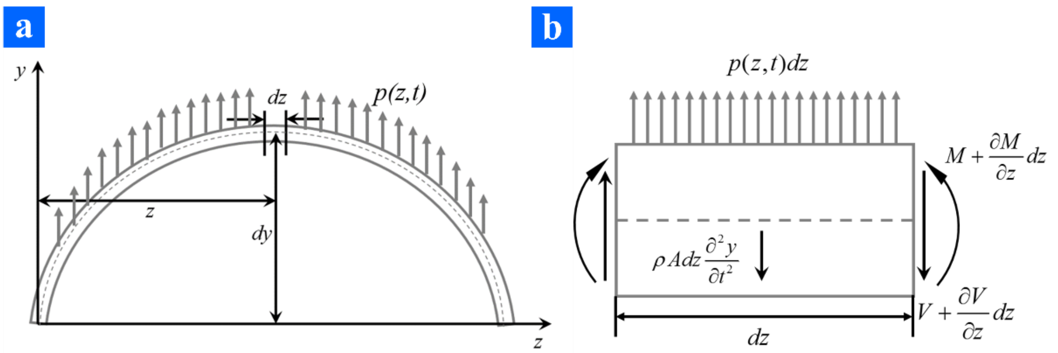

Figure S1 Dynamic model of beam bending vibration

$$EI\frac{\partial^4 Y(z,t)}{\partial z^4}+\rho A\frac{\partial^2 Y(z,t)}{\partial t^2}=f(z,t) \tag{S1}$$

Where, *I* represents the moment of inertia of the resonant beam, *E* represents the Young's modulus of the ZnO resonant beam, *ρ* represents the density of the resonant beam, and *A* represents the cross-sectional area of the resonant beam.

According to the principle of superposition of vibration modes, the lateral displacement *Y(z, t)* can be written as:

$$Y(z,t) = y(z)q(t) \tag{S2}$$

Where *y(z,t)* is the lateral bending mode function, *q(t)* is the propagation equation of the transverse wave, and *q(t)* is harmonic. Substituting into equation S1 gives:

$$EI\frac{y''''(z)}{y(z)} - \frac{\rho A q''(t)}{q(t)} = \rho A \omega^2 \tag{S3}$$

Write the mode shape and time function into independent equations:

$$\begin{gathered} EIy''''(z) - \rho A \omega^2 y(z) = 0 \\ q''(t) + \omega^2 q(t) = 0 \end{gathered} \tag{S4}$$

The first equation in equation (S4) is a fourth-order differential equation, and its general solution is:

$$y(z) = Ce^{sz} \tag{S5}$$

Substituting the mode shape function gives:

$$s^4 - \frac{\rho A}{EI}\omega^2 = 0 \tag{S6}$$

Let:

$$\beta^4 = \frac{\rho A \omega^2}{EI} \tag{S7}$$

The four solutions to the one-variable fourth-degree equation of equation (S7) are:

$$s_{1,2,3,4} = \pm\beta i, \pm\beta \tag{S8}$$

Therefore, the solution to the vibration mode function in equation (S4) is:

$$\begin{aligned} y(z) &= C_1 e^{-i\beta z} + C_2 e^{i\beta z} + C_3 e^{-\beta z} + C_4 e^{\beta z} \\ &= A\sin\beta z + B\cos\beta z + C\sinh\beta z + D\cosh\beta z \end{aligned} \tag{S9}$$

Where A, B, C, D are unknowns, and due to the fixed support at both ends of the ZnO nanobeam resonator, according to the boundary conditions of the fixed beam, the following equations can be obtained about the unknowns A, B, C, and D:

$$y(0)=0, y'(0)=0, y(L)=0, y'(L)=0 \tag{S10}$$

$$\begin{pmatrix} 0 & 1 & 0 & 1 \\ \beta & 0 & \beta & 0 \\ \sin(\beta L) & \cos(\beta L) & \sinh(\beta L) & \cosh(\beta L) \\ \beta\cos(\beta L) & -\beta\sin(\beta L) & \beta\cosh(\beta L) & \beta\sinh(\beta L) \end{pmatrix}\begin{pmatrix} A \\ B \\ C \\ D \end{pmatrix}=\begin{pmatrix} 0 \\ 0 \\ 0 \\ 0 \end{pmatrix} \tag{S11}$$

Since the coefficient matrix of equation (S11) cannot be all 0, the determinant of the coefficient matrix is 0, which is expressed as:

$$\begin{vmatrix} 0 & 1 & 0 & 1 \\ \beta & 0 & \beta & 0 \\ \sin(\beta L) & \cos(\beta L) & \sinh(\beta L) & \cosh(\beta L) \\ \beta\cos(\beta L) & -\beta\sin(\beta L) & \beta\cosh(\beta L) & \beta\sinh(\beta L) \end{vmatrix}=0 \tag{S12}$$

From equation (S12), the transverse free vibration frequency equation of the ZnO nanobeam can be obtained as:

$$cos(\beta L)\cosh(\beta L)=1 \tag{S13}$$

Equation (S13) is a transcendental equation. The numerical solution of βiL can be obtained by numerical calculation methods, which are $\beta_1 L$=4.730, $\beta_2 L$=7.853, $\beta_3 L$=10.996. Combined with equation (S7), the resonant frequency of the ZnO nanobeam in the transverse direction can be obtained as follows:

$$f_0=\frac{(\beta_i L)^2}{2\pi}\sqrt{\frac{EI}{\rho A L^2}} \tag{S14}$$

The ZnO nanobeam used in the accelerometer structure has a hexagonal cross section, and its moment of inertia and cross-sectional area are:

$$I=\frac{5\sqrt{3}d^4}{256}, A=\frac{3\sqrt{3}d^2}{8} \tag{S15}$$

Where d is twice the edge length of the beam section. Under the microscope, the cross-section of the ZnO nanobeam is nearly circular. For ease of measurement, the parameter d in the following text refers to the diameter of the nanobeam, and L is the length of the resonant beam. Combined with equations (S8), (S14), and (S15), the first-order bending fundamental frequency of the ZnO nanobeam in free vibration can be obtained as follows:

$$f_0 = \frac{(\beta_i L)^2}{2\pi}\sqrt{\frac{EI}{\rho A L^2}} \tag{S16}$$

Then, the vibration mode function can be written as:

$$y(z) = \cos\beta z - \cosh\beta z - \frac{\cos\beta L - \cosh\beta L}{\sin\beta L - \sinh\beta L}(\sin\beta z - \sinh\beta z) \tag{S17}$$

Sensitivity of the resonant accelerometer

Figure S2 shows the dynamic model of a vibrating beam subjected to axial force. When a resonant beam is subjected to an axial force, the vibration equation is expressed as:

$$EI\frac{\partial^4 Y(z,t)}{\partial z^4} + N\frac{\partial^2 Y(z,t)}{\partial z^2} + \rho A\frac{\partial^2 Y(z,t)}{\partial t^2} = f(z,t) \tag{S18}$$

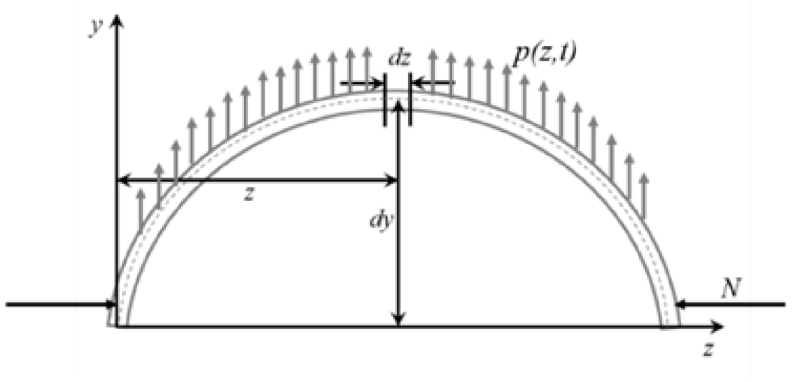

Figure S2 Dynamic model of vibration beam subjected to axial force

where N is the equivalent axial force on the resonant beam in the accelerometer under acceleration load, combined with equation (S2), equation (S18) can be written as:

$$EI\frac{\partial^4 y}{\partial z^4}q + N\frac{\partial^2 y}{\partial z^2}q + \rho A y\frac{\partial^2 q}{\partial t^2} = f(z,t) \tag{S19}$$

Integrating equation (S19) over the entire resonant beam, we obtain:

$$(\int_L^0 \rho A y^2 dz)\ddot{q} + (\int_L^0 EI \frac{\partial^4 y}{\partial z^4} y dz + \int_L^0 N \frac{\partial^2 y}{\partial z^2} y dz) q = 0 \tag{S20}$$

From the above equation, the maximum elastic potential energy and maximum kinetic energy of the resonant beam are:

$$\begin{cases} U_{\max} = \frac{1}{2}\int_L^0 EI \frac{\partial^4 y}{\partial z^4} y dz + \frac{1}{2}\int_L^0 N \frac{\partial^2 y}{\partial z^2} y dz \\ T_{\max} = \frac{1}{2}\omega^2 \int_L^0 \rho A y^2 dz \end{cases} \tag{S21}$$

According to the law of conservation of energy, the natural angular frequency of the resonant beam under axial force can be obtained as:

$$\omega = \sqrt{\frac{\int_L^0 EI \frac{\partial^4 y}{\partial z^4} y dz + \int_L^0 N \frac{\partial^2 y}{\partial z^2} y dz}{\int_L^0 \rho A y^2 dz}} \tag{S22}$$

Substituting the vibration mode equation for free vibration, we obtain the frequency of the resonant beam as:

$$\begin{cases} f = \frac{\omega}{2\pi} = f_0 \sqrt{1 + \frac{NL^2}{4\pi^2 EI}} = f_0 \sqrt{1 + \frac{0.749 NL^2}{Ed^4}} \\ f_0 = \frac{(4.73)^2 d}{2\pi L^2} \sqrt{\frac{5E}{96\rho}} \end{cases} \tag{S23}$$

Expanding equation (S23) using Taylor series, we obtain:

$$f = f_0(1 + \frac{1}{2}[\frac{0.749L^2}{Ed^4}N] - \frac{1}{8}[\frac{0.749L^2}{Ed^4}N]^2 + \frac{1}{16}[\frac{0.749L^2}{Ed^4}N]^3 - \frac{5}{128}[\frac{0.749L^2}{Ed^4}N]^4 + o(N^5)) \tag{S24}$$

When the accelerometer is subjected to a 1g acceleration, the resonant beam subjected to axial tension has an increased stiffness and bending vibration frequency, and the resonant beam subjected to axial compression has a decreased stiffness and bending vibration frequency. The frequency difference between the resonant beams on the left and right sides is:

$$\Delta f = f_1 - f_2 = f_0(\frac{0.749L^2}{Ed^4}N + \frac{1}{8}[\frac{0.749L^2}{Ed^4}N]^3 + o(n^5)) \tag{S25}$$

Ignoring the influence of higher-order small quantities on sensitivity, the sensitivity expression is therefore:

$$\Delta f = f_0 \frac{0.749L^2}{Ed^4} N + \frac{1}{8} f_0 [\frac{0.749L^2}{Ed^4} N]^3 \quad (S26)$$

The first part of equation (S26) is the linear sensitivity of the accelerometer, and the latter part affects the nonlinearity of the accelerometer sensitivity. Based on the force-sensitive characteristic of the resonant accelerometer, the sensitivity is affected by the size of the axial force, and it increases with the increase of the axial force. Since the sensitivity expression has a third-order nonlinearity term, it can be ignored when the axial force is small. When the axial force is large, it seriously affects the linearity of the accelerometer sensitivity, limiting the range of the accelerometer. Therefore, a balance must be struck between sensitivity, range, and the mutually suppressive effect of frequency nonlinearity. When the accelerometer is operating, the acceleration load acts on the mass block, and the small stiffness of the flexible neck on the supporting beam causes an equivalent bending moment that causes the mass block to rotate around the flexible beam, and the rotation angle is related to the size of the flexible beam and the mass block. Therefore, the parameter *N*, which represents the axial force on the nanobeam, is related to the structure, size, mass block size, flexible neck structure, and distance between the two beams of the accelerometer, all of which have a significant impact on the sensitivity of the accelerometer.

Note S2 Design of the notched flexure

The effect of the notched flexure size on the resonant performance of the ZnO nanobeam with a diameter of 500 nm and a length of 50 μm was analyzed by applying an acceleration load of 1g in the x-direction and y-direction, respectively. The results are shown in Figure S3c and Figure S3d. As shown in Figure S3, the resonant frequency of the ZnO nanobeam was changed greatly and the difference between the symmetrically distribute ZnO nanobeams was bigger as the width of the notched flexure narrows, indicating that the sensitivity of the accelerometer

would be higher as the width of the notched flexure was smaller. Figure S3d shows the resonant frequency of the ZnO nanobeam under a 1g load acceleration in the non-sensitive direction for different widths of the notched flexure. As shown in Figure S3d, the resonant frequency of the ZnO nanobeam increases as the notched flexure width increases and the difference in resonance frequency in the non-sensitive direction decreases with an increase in the notched flexure's width. Additionally, when the notched flexure width exceeds 2 μm, the frequency difference fluctuation becomes small. It is worth noting that the resonance frequency difference in the non-sensitive direction is significantly smaller than that in the sensitive direction. Therefore, the width of the notched flexure, as a key parameter of the accelerometer motivating component, plays a crucial role in improving the sensitivity of the accelerometer. Considering the processing technology, a rectangle notched flexure with a width of 3 μm and a length of 2 μm was proposed.

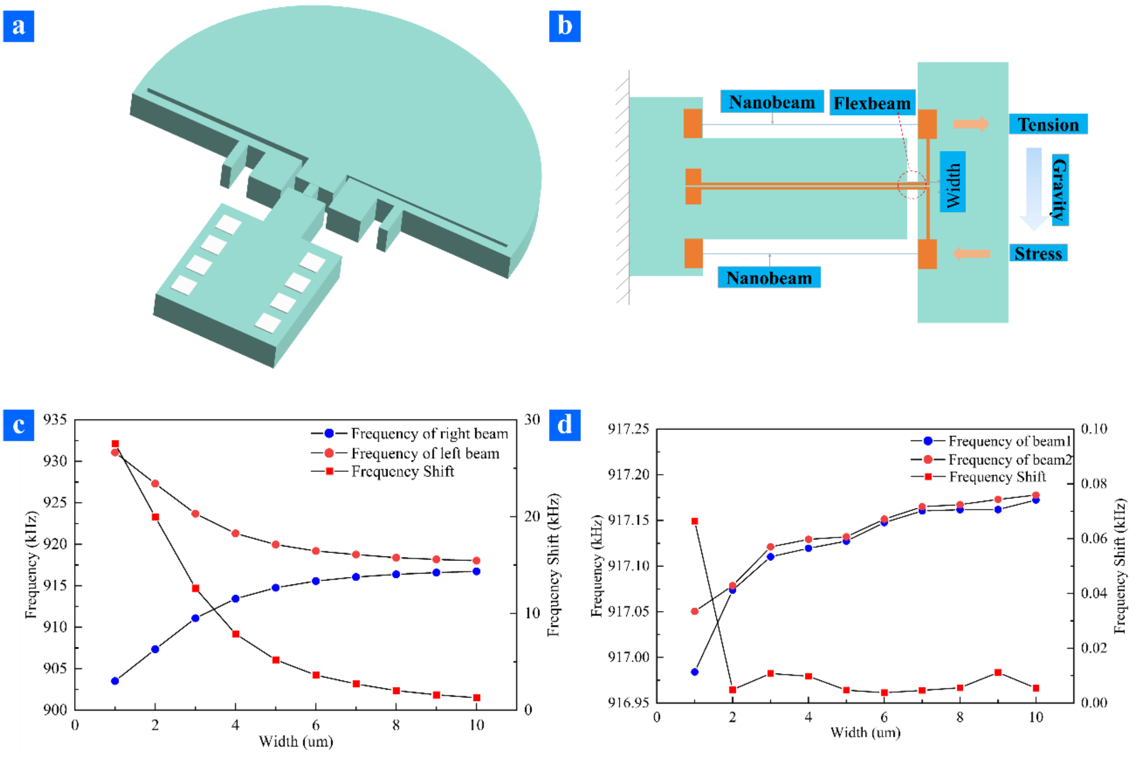

Figure S3. The schematic diagram of notched flexure, (a) the total structure of the differential resonant accelerometer, (b) The detail structure of the notched flexure, (c) the relationship

between resonant frequency and the width of the notched flexure in sensitive direction, (d) the relationship between resonant frequency and the width of the notched flexure in non-sensitive direction

Note S3 Design of the microleverage

To enhance the sensitivity of the accelerometer, a microleverage mechanism was implemented. According to the position of the pivot beam, input beam, and output beam of the microleverage, the microleverage was classified into three types. The first type of microleverage has a flexible support beam between the input beam and the output beam. When the dynamic arm is greater than the resistance arm, this structure can simultaneously amplify inertial forces and micro-displacements, and also has the function of changing the direction of inertial forces and micro-displacements. The second type microleverage mechanism has input and output beams on the same side of the support beam, with the output beam closer to the support beam. This type of structure is mainly used to amplify inertial forces. The third type microleverage mechanism has input and output beams on the same side of the support beam, with the input beam closer to the support beam. This type of structure is mainly used to amplify micro-displacements. Herein, the first type of microleverages mechanism was adopted to improve the sensitivity of the accelerometer and facilitate electrodes fabrication. The structure of the miceoleverage was optimized with FEA method which evaluated its performance by the deformation of the ZnO nanobeam. The results were displayed in Figure S4d and Figure S4e. The deformation of the ZnO nanobeam initially increased and then decreased as the distance between the support beam and output beam increased from 6 to 120 μm, with the maximum deformation occurring when the distance was 36 μm, as shown in Figure S4d. As shown in Figure S4e, it is evident that the narrower the width of the support beam, the larger the deformation of the ZnO nanobeam. However, if the width of the support was too small, the stability of the microleverage may be

compromised. Hence, the support beam of the microleverage mechanism was determined to be 3 μm with a distance of 36 μm from the output beam.

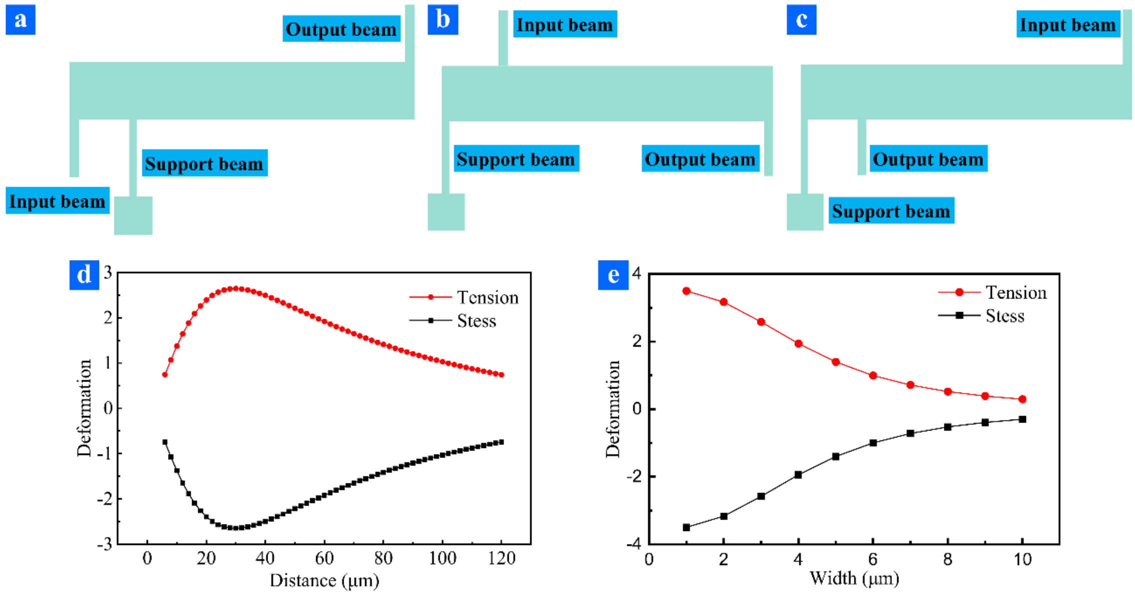

Figure S4. The microleverage structure of accelerometer (a) the first type microleverage structure, (b) the second type microleverage structure, (c) the third type microleverage structure, (d) the optimization of the microleverage support beam position, (e) the optimization of the microleverage support beam width.

Note S4 Design of the unloading grooves anchor

The differential resonant accelerometer can suppress the majority of the resonant frequency shifts caused by temperature, pressure and other factors. The size and properties of the two ZnO nanowires, however, can't guaranteed to be completely consistent. So the resonant frequency fluctuations caused by temperature, pressure and other factors can't eliminate completely. In the configuration involving a silicon structure, a ZnO nanowire, and a metal electrode in direct contact. Consequently, owing to distinct thermal expansion coefficients among these three materials, internal stresses are introduced as a consequence of thermal expansion. Specifically, axial stress induced by thermal expansion directly impacts the resonant frequency of the ZnO nano-resonator. To effectively address this phenomenon, a differential design of device

structures and parameter optimization could eliminate the majority of the frequency shifts caused by structural expansions and internal stresses. Furthermore, the design of an anchor structure that releases stress promptly can also alleviate the impact of thermal stress on the ZnO nano-resonator. Figure S5 illustrates schematic diagrams depicting rectangular anchor points and unloading anchor points, respectively.

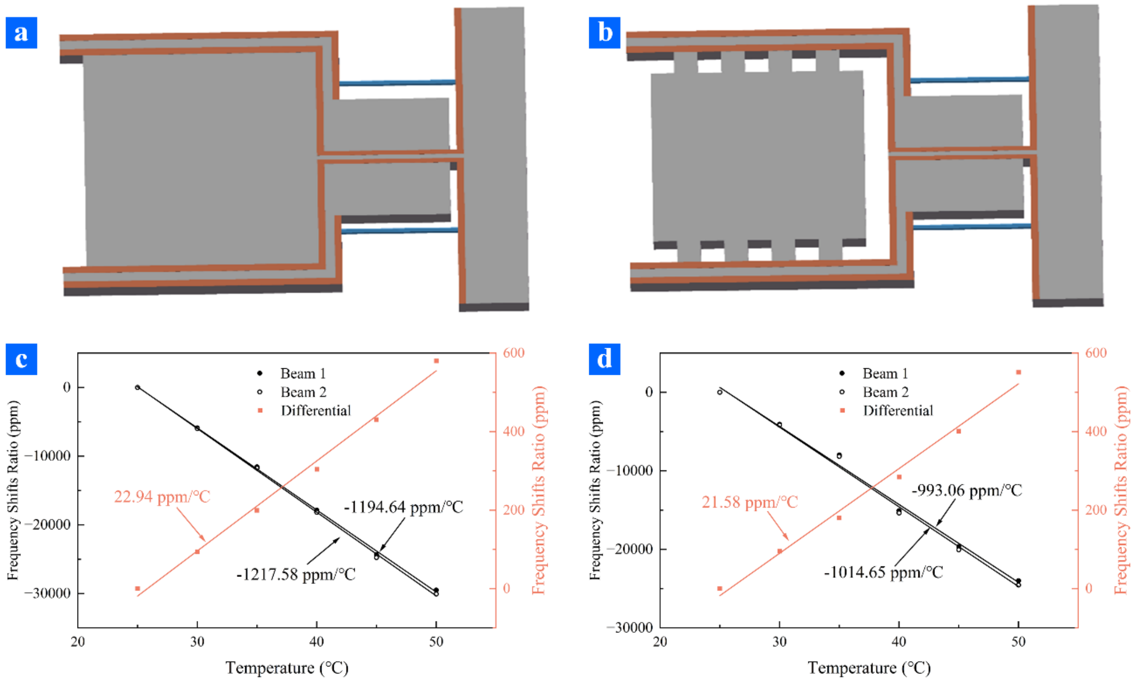

Figure S5. The anchor structure of accelerometer. (a) The rectangle anchor structure. (b) The unloading anchor structure. The resonant frequency shifts of the rectangle anchor structure (c) and the unloading anchor structure (d).

To investigate the influence of thermal stress on the three anchor points, the resonant frequency shifts of the ZnO resonant nano-accelerometer were analyzed using Finite Element Analysis (FEA). The operational temperature ranged from 25°C to 50°C. The Young's modulus values for ZnO and Si were assumed to be 150 GPa and 120 GPa, respectively. The coefficients of thermal expansion for ZnO and Si were obtained from sources such as D. Taylor *et al. Transactions and Journal of the British Ceramic Society*, v83, No. 1, p1, R.R. Rebber *et al. Journal of Applied Physics*, v41, No. 13, and C.A. Swenson *et. al*, *Journal of Physical and*

Chemical Reference Data, v12, No. 2, within the specified temperature range. The accelerometer resistibility to temperature change was evaluated with the ratio of frequency shifts caused by temperature change to resonant frequency of accelerometers at 25 ℃. As illustrated in Figure S5, the differentially designed architecture successfully mitigated the majority of frequency shifts attributed to temperature variations. Furthermore, the structure with unloading grooves contributed to the stability improvement by facilitating the release of stress resulting from temperature changes.

Note S5 Stability of ZnO nano-accelerometer

The frequency stability of ZnO nano-resonator was one of the key metrics to reveal the noise level of the device, which determines the resolution of the ZnO nano-accelerometer. For ZnO nano-resonator, the experiment Allan deviation of ZnO nano-resonators was measured in open loop measurement recording of the phase variation of electrical signal at resonant frequency. The ZnO nano-resonator was driven at its resonant frequency (716 kHz) with a time constant of 1 ms for 1800s. The experimental result was shown in Figure S6. For acceleration measurement has to be focused on short integration time. Typically, we achieved an Allan deviation about of 10^{-6} for $\tau = 0.25$ s at room temperature.

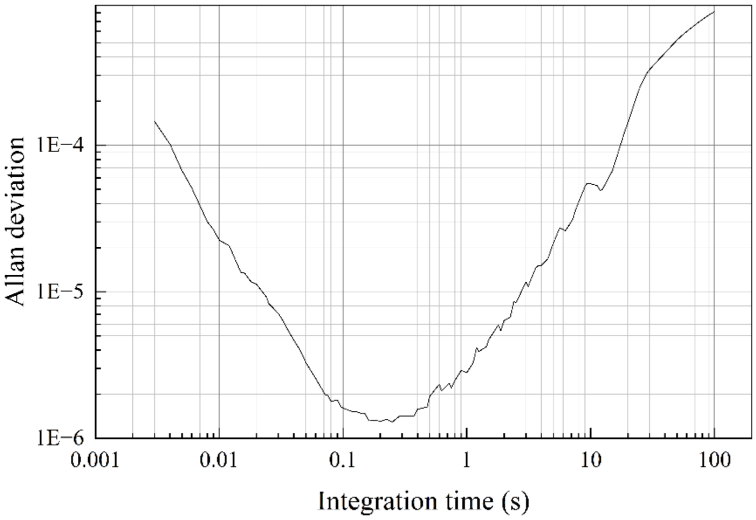

Figure S6. Allan deviation of ZnO nano-resonator measured in open loop condition.

The open loop measurement method was able to calibrate the sensitivity of the ZnO nano-accelerometer, however, it was not suitable for the practical application. It was expected to build a closed loop to trace the resonant frequency in real time. So the feedback loop was prepared to trace the resonant frequency of the accelerometers with a lock-in amplifier. As displayed in Figure 3b, an AC voltage was applied on the source, and a combination of AC voltage and DC voltage was applied on the gate to actuate the ZnO nano-resonator into high frequency vibration motion. Meanwhile, a mixing principle based on lock-in amplifier was employed to read the frequency response of the ZnO nano-resonator. To trace the resonant frequency, the current value was employed, keeping I around a reference value I_{ref} by varying f. As displayed in Figure S7, when I remains between I_{min} and I_{max}, the driving frequency was not changed and, accordingly, the shift of the resonant frequency was calculated from I and the slope of the frequency around I_{ref}. The feedback time can be made as low as 50 ms. The feedback is interrupted repeatedly (typically every 600 s) during ~10 s for a control of the lineshape of the current-frequency (I as a function of f): if the resonant lineshape significantly differs from that measured previous, the recorded data were discarded.

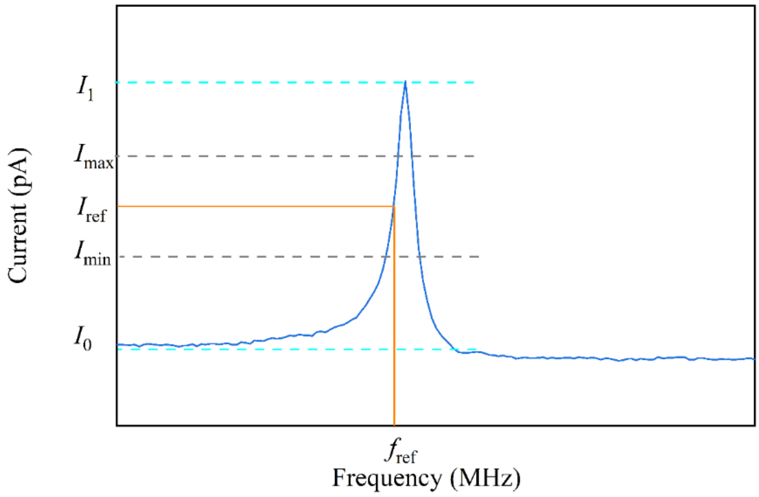

Figure S7. Schematic of the response of the mixing current as a function driving frequency

Here, we indicated typical parameters used in the feedback loop. We often set the reference current to $I_0 + 0.5(I_1 - I_0)$ on the low frequency part of the current-frequency curve, I_1 being

the highest current of the curve; but I_{ref} can also be lowered down to $I_0 + 0.3(I_1 - I_0)$ where the slope of the curve was steeper. We set the thresholds I_{max} and I_{min} so that (I_{max} - I_{min}) / I_0 is typically 0.5 (we also tried values between 0.1 and 0.7). The current range (I_{max} - I_{min}) corresponds to a frequency range that is typically from 1 kHz to 10 kHz, which depends on the bandwidth of the resonators. For the bias instability measurement of the accelerometer, the feedback time was set as 200 ms. The ZnO resonant nano-accelerometer was mounted at 0g position, and working temperature was about 25 °C. The resonant frequency differential trace as function of time was displayed in Figure S8.

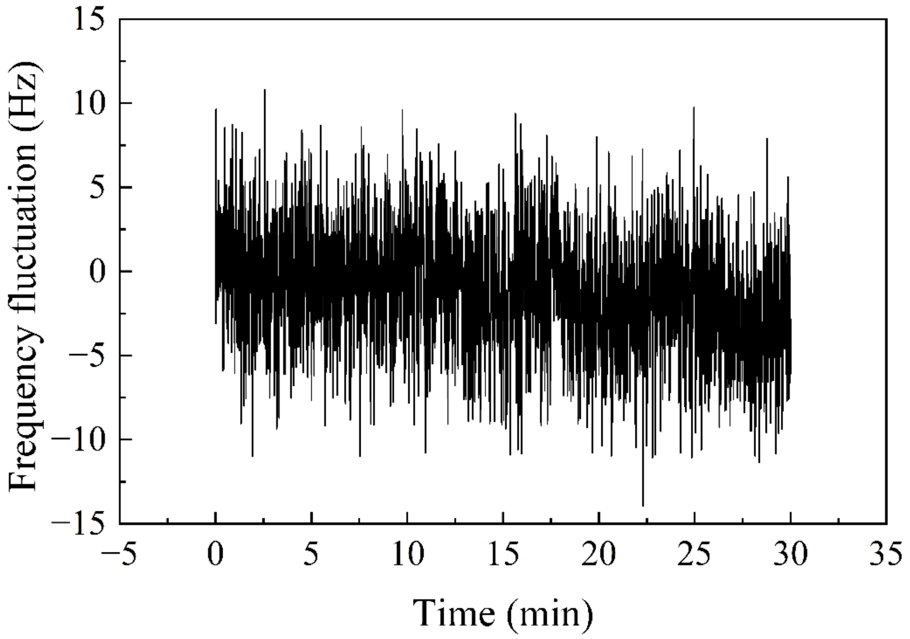

Figure S8 The resonant frequency differential trace as function of time

Note S6 Sensitivity reproducibility of ZnO resonant nano-accelerometers

The sensitivity of the accelerometer was checked many times, and the longest interval time was over a month. The sensitivity was measured to be 16.298 kHz/g, demonstrating a good single device reproducibility. In addition, over 20 ZnO resonant nano-accelerometers of were prepared by optical microscope manipulation and FIB technology. The sensitivity of the devices was measured as introduced in the manuscript, majority of them were up to 10 kHz/g, which benefited from the ZnO nano-resonators.

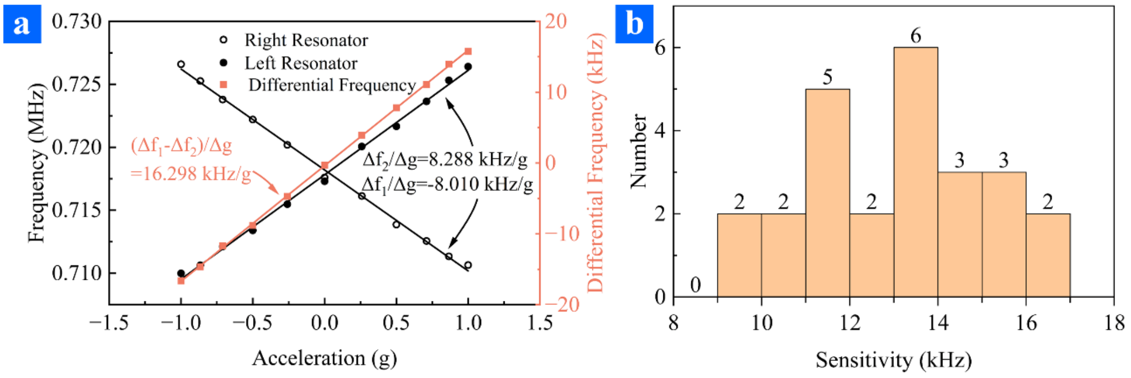

Figure S9 (a) The sensitivity of ZnO resonant nano-accelerometer after a month. (b) Sensitivity statistical diagram of ZnO resonant nano-accelerometers

Note S7 The comparison of our device and other reported resonant accelerometers

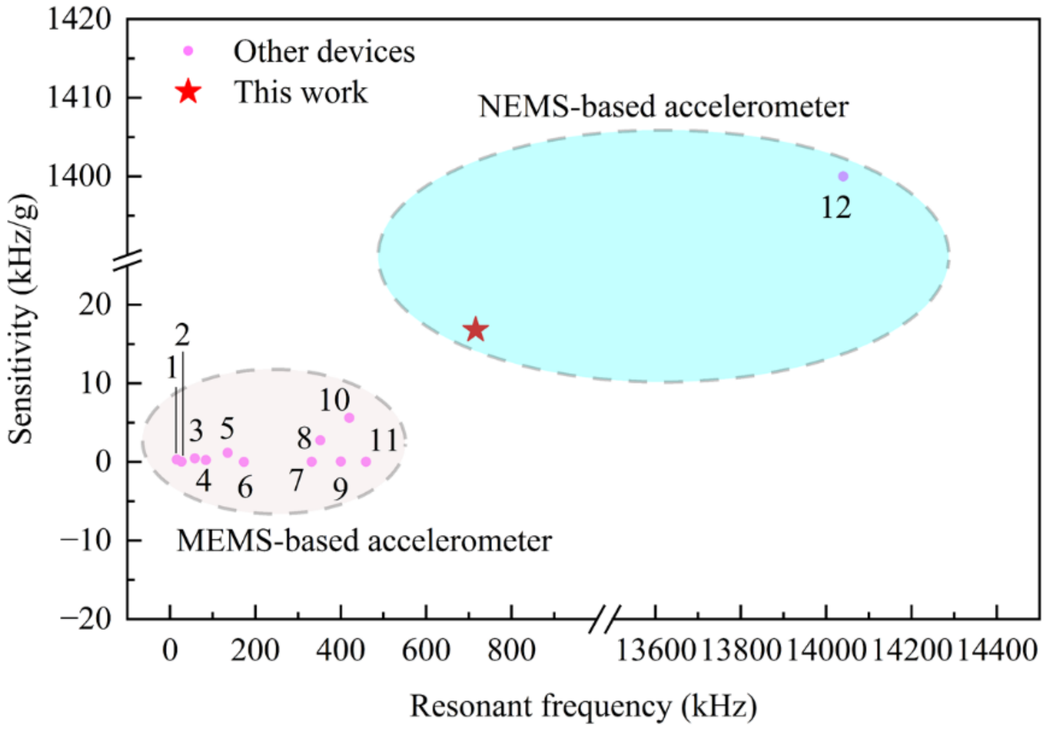

Figure S10 The sensitivity and resonant frequency of this work and other resonant accelerometers.

Table S1. Comparison of the resonant frequency, sensitivity and relative sensitivity of previous devices and our device

Number	Reference	Sensitivity (kHz)	Resonant frequency (kHz)	relative sensitivity (ppm)
1	Xie *et al.*	0.2975	16.061	18520
2	Yang *et al.*	0.052	27	1930
3	Comi *et al.*	0.455	58	7840
4	Caspani *et al.*	0.25	84	2980
5	Wang *et al.*	1.1533	135	8540
6	Seshia *et al.*	0.017	173	98.2659
7	Gabriele *et al.*	0.0181	332	54.5181
8	Zhao *et al.*	2.752	352.2	7810
9	Aikele *et al.*	0.07	400	175
10	Zou *et al.*	5.61	420	13360
11	Pinto *et al.*	0.022	459	47.9303
12	Maro *et al.*	1400	14040	97000
	This paper	16810	716	23480

Note S8 Characterization of ZnO nanowires

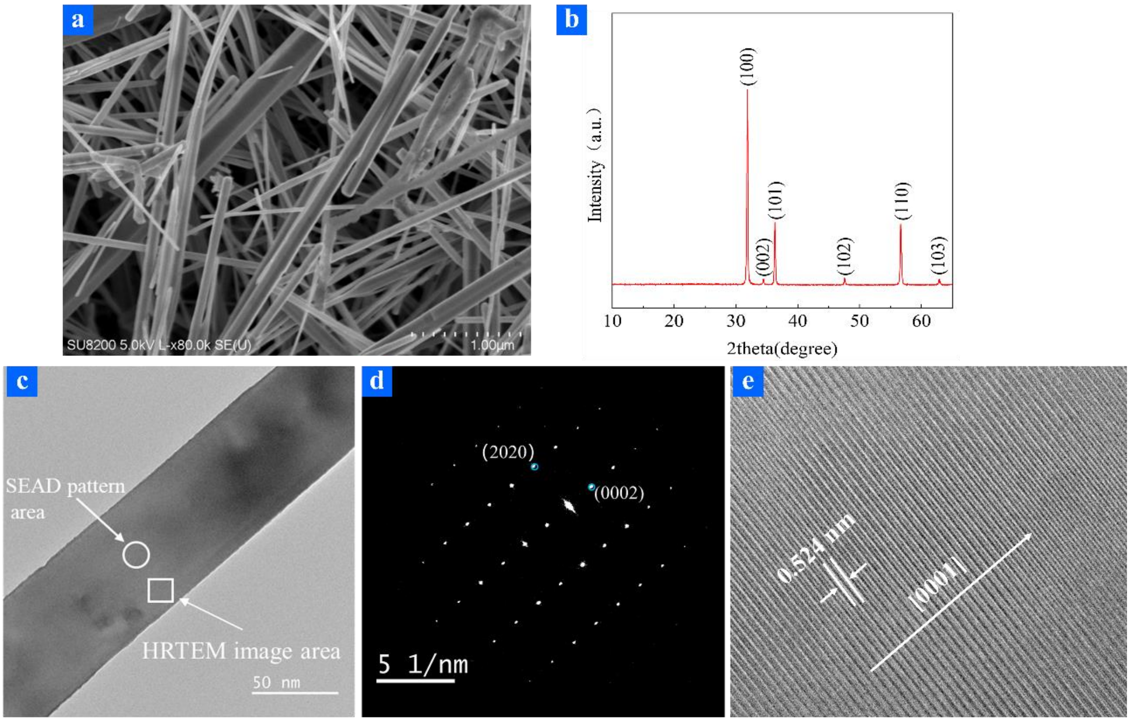

Figure S11. The SEM image(a) and X-ray diffraction (XRD) patterns (b) of ZnO nanowires (c) low magnification TEM image of ZnO nanowire, (d) the electron diffraction (SAED) pattern of ZnO nanowire at the white circle in (c), (e) HRTEM image of ZnO nanowire at the white square in (c)

Note S9 Fabrication of ZnO nano-resonator

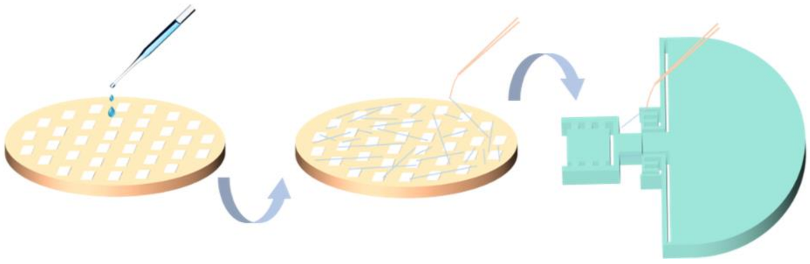

Figure S12. Schematic diagram of optical microscope nanomanipulation.

Note S10 Simulation model for ZnO nano-resonator and accelerometers

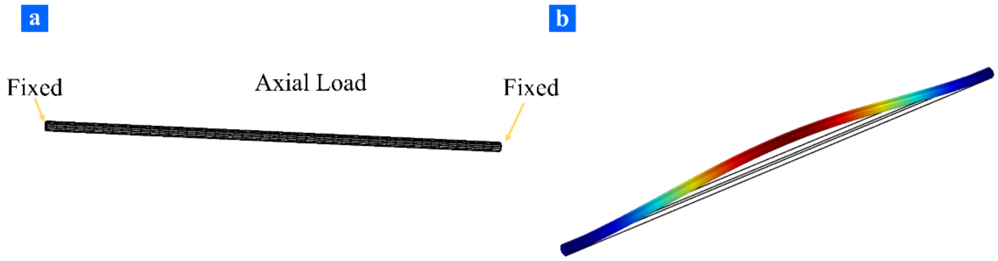

Figure S13. Simulation model for ZnO nanobeam.

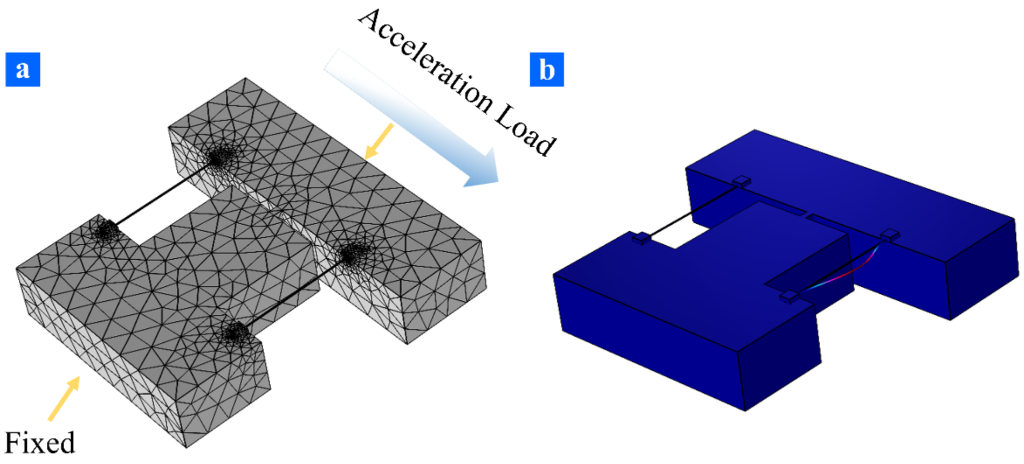

Figure S14. Simulation model for notched flexure.

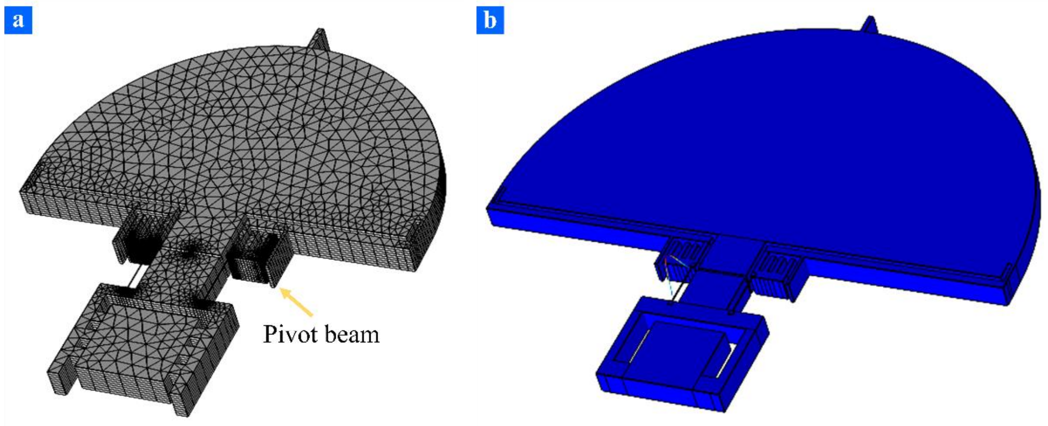

Figure S15. Simulation model for microleverages.

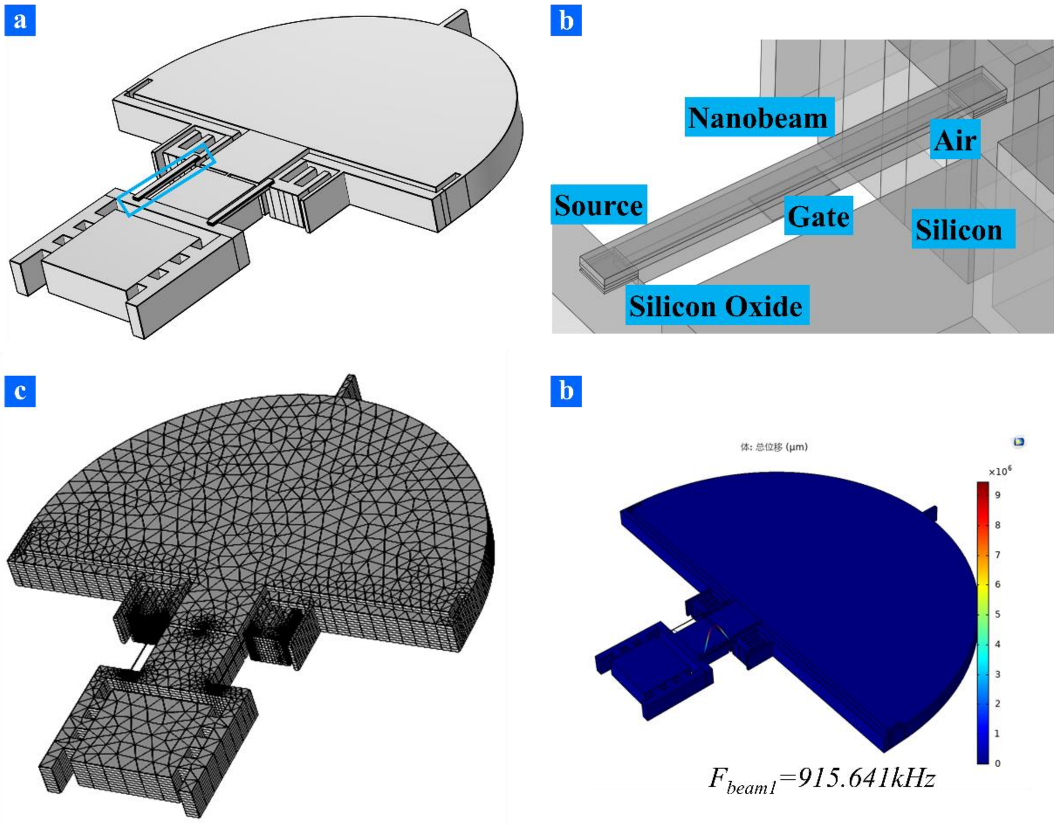

Figure S16. Simulation model for the sensitivity of the accelerometer.

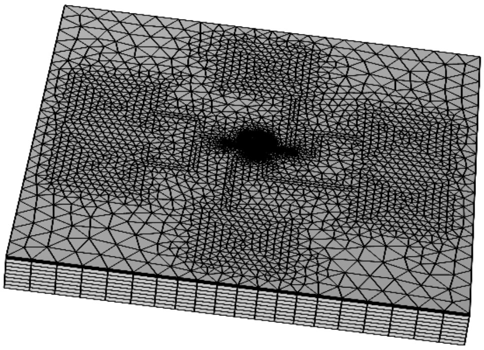

Figure S17. Simulation model for the accelerometer’s resonant frequency shifts caused by temperature change.

Supplementary References

1 T. Miani, *et al.*, Resonant accelerometers based on nanomechanical piezoresistive transduction, 34th IEEE MEMS, Electr Network, 192-195, (2021)

2 Comi, C. *et al.* A Resonant Microaccelerometer With High Sensitivity Operating in an Oscillating Circuit. *Journal of Microelectromechanical Systems* 19, 1140-1152, (2010).

3 Caspani, A., Comi, C., Corigliano, A., Langfelder, G. & Tocchio, A. Compact biaxial micromachined resonant accelerometer. *Journal of Micromechanics and Microengineering* **23**, 1-11, (2013).

4 Aikelea, M. *et al.* Resonant accelerometer with self-test. *Sensors and Actuators A* **92**, 161-167, (2001).

5 Seshia, A. A. *et al.* A vacuum packaged surface micromachined resonant accelerometer. *Journal of Microelectromechanical Systems* **11**, 784-793, (2002).

6 Pinto, D. *et al.* A Small and High Sensitivity Resonant Accelerometer. *Procedia Chemistry* **1**, 536-539, (2009).

7 Vigevani, G., Goericke, F. T., Pisano, A. P., Izyumin, I. I. & Boser, B. E. in *2012 IEEE International Frequency Control Symposium Proceedings.*

8 Yang, B., Zhao, H., Dai, B. & Liu, X. A new silicon biaxial decoupled resonant micro-accelerometer. *Microsystem Technologies* **21**, 109-115, (2014).

9 Wang, S., Wei, X., Zhao, Y., Jiang, Z. & Shen, Y. A MEMS resonant accelerometer for low-frequency vibration detection. *Sensors and Actuators A: Physical* **283**, 151-158, (2018).

10 Pandit, M. S. *et al.* An Ultra-High Resolution Resonant MEMS Accelerometer. *2019 IEEE 32nd International Conference on Micro Electro Mechanical Systems (MEMS)*, 664-667, (2019).

11 Zhao, C. *et al.* A Resonant MEMS Accelerometer With 56 ng Bias Stability and $98ng/Hz^{1/2}$ Noise Floor. *Journal of Microelectromechanical Systems* **28**, 324-326, (2019).

12 Ding, H., Wu, C. & Xie, J. A MEMS Resonant Accelerometer With High Relative Sensitivity Based on Sensing Scheme of Electrostatically Induced Stiffness Perturbation. *Journal of Microelectromechanical Systems* **30**, 32-41, (2021).
